# Supplementary material for: Distinct mechanisms of inhibition of Kv2 potassium channels by tetraethylammonium and RY785
Source: bioRxiv. 2024 Jul 25:2024.07.25.605170. Preprint. [Version 1] doi: 10.1101/2024.07.25.605170 (PMC11451595; doi:10.1101/2024.07.25.605170)
Supplement: Supplement 1 [file media-1.pdf]

Distinct mechanisms of inhibition of Kv2 potassium channels by tetraethylammonium and RY785

Supplementary Information

Shan Zhang[1], Robyn Stix[1,2], Esam A. Orabi[1], Nathan Bernhardt[1], José D. Faraldo-Gómez[1*]

[1]Theoretical Molecular Biophysics Laboratory,
National Heart, Lung and Blood Institute,
National Institutes of Health, Bethesda, MD

[2]Molecular and Cell Biology Graduate Program,
Johns Hopkins University, Baltimore, MD

*Correspondence should be addressed to: jose.faraldo@nih.gov

July 25th, 2024

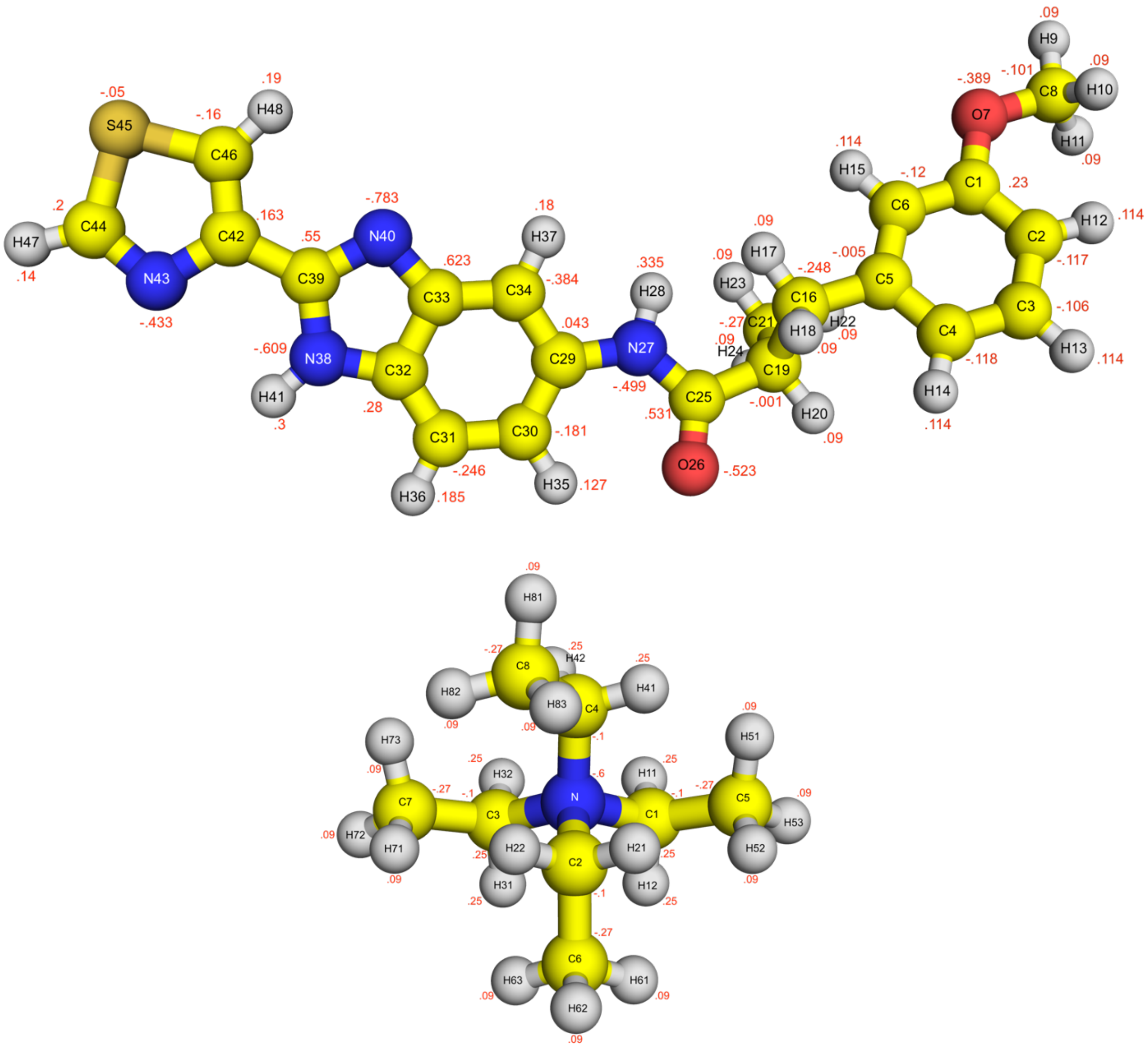

Figure S1. Chemical structures of RY785 and tetraethylammonium. Atom names and partial electronic charges are indicated.

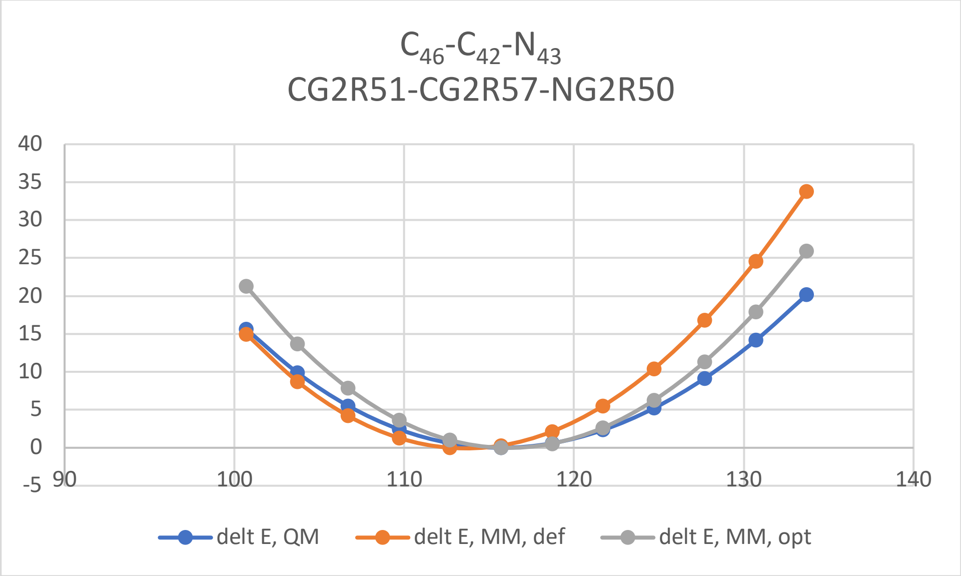

Figure S2. Potential-energy curve for the $C_{46}C_{42}N_{43}$ angle between 100° and 135°, calculated with MP2/6-31G(d) (blue) and with default CGengFF (orange) or with our optimized forcefield (gray). The Y axis shows the energy (in kcal/mol) relative to the lowest-energy conformer.

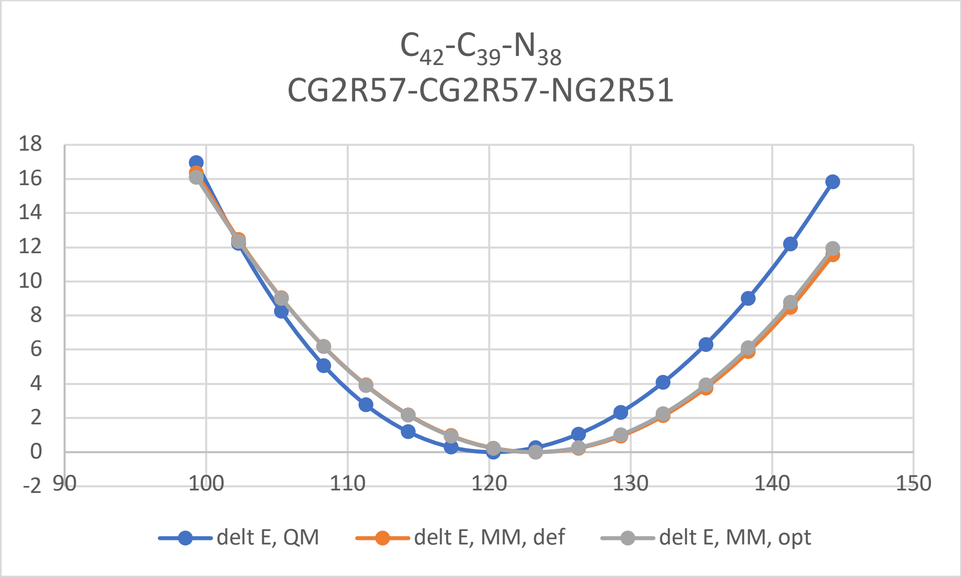

Figure S3. Potential-energy curve for the $C_{42}C_{39}N_{38}$ angle between 90° and 145°, calculated with MP2/6-31G(d) (blue) and with default CGengFF (orange) or with our optimized forcefield (gray). The Y axis shows the energy (in kcal/mol) relative to the lowest-energy conformer.

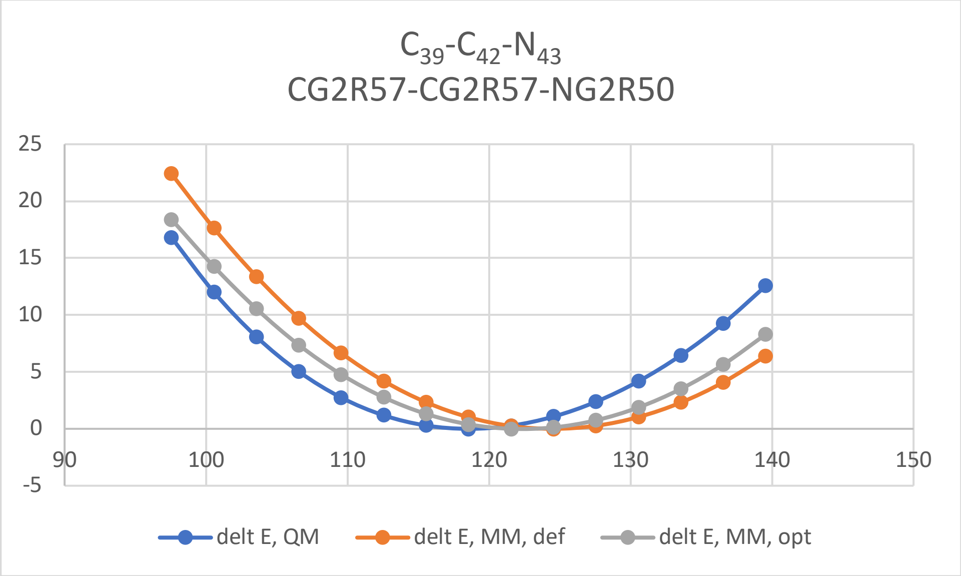

Figure S4. Potential-energy curve for the $C_{39}C_{42}N_{43}$ angle between 95° and 140°, calculated with MP2/6-31G(d) (blue) and with default CGengFF (orange) or with our optimized forcefield (gray). The Y axis shows the energy (in kcal/mol) relative to the lowest-energy conformer.

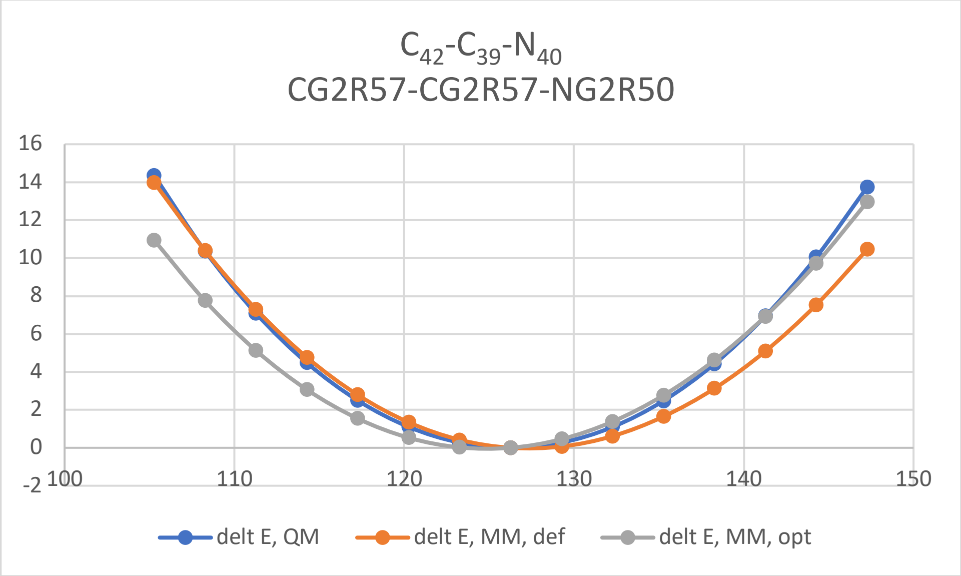

Figure S5. Potential-energy curve for $C_{42}C_{39}N_{40}$ angle between 105° and 150°, calculated with MP2/6-31G(d) (blue) and with default CGengFF (orange) or with our optimized forcefield (gray). The Y axis shows the energy (in kcal/mol) relative to the lowest-energy conformer.

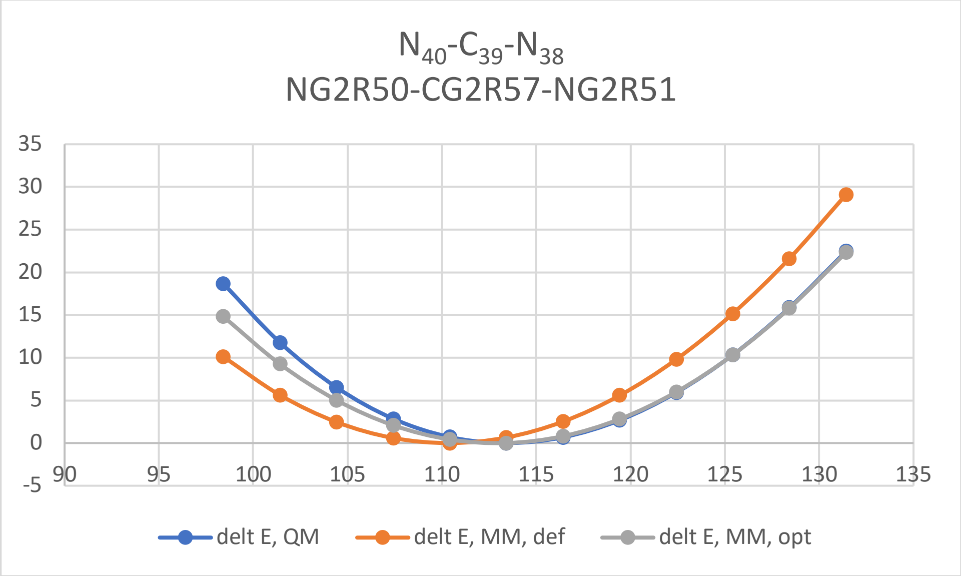

Figure S6. Potential-energy curve for the $N_{40}C_{39}N_{38}$ angle between 95° and 135°, calculated with MP2/6-31G(d) (blue) and with default CGengFF (orange) or with our optimized forcefield (gray). The Y axis shows the energy (in kcal/mol) relative to the lowest-energy conformer.

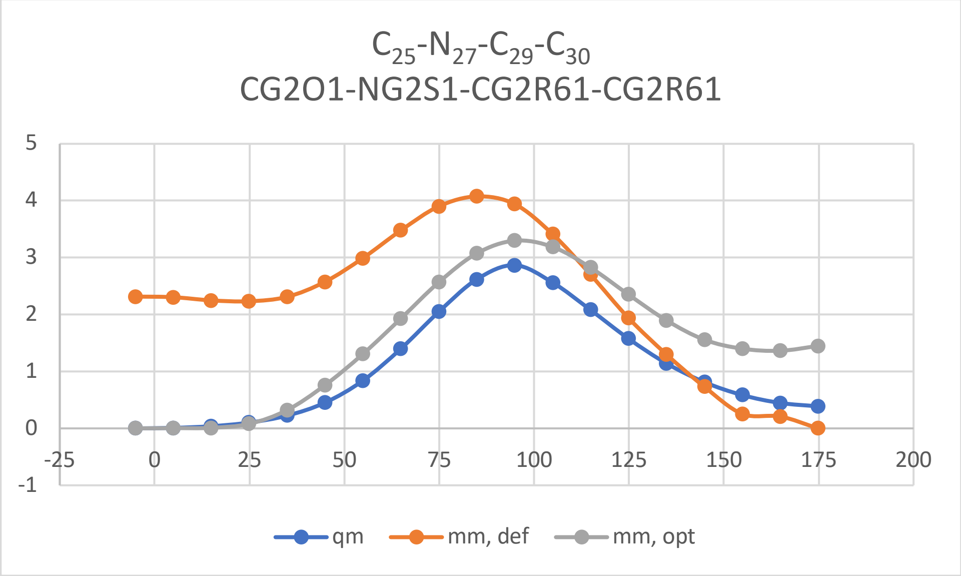

Figure S7. Potential-energy curve for the $C_{25}N_{27}C_{29}C_{30}$ angle between 0° and 180°, calculated with MP2/6-31G(d) (blue) and with default CGengFF (orange) or with our optimized forcefield (gray). The Y axis shows the energy (in kcal/mol) relative to the lowest-energy conformer.

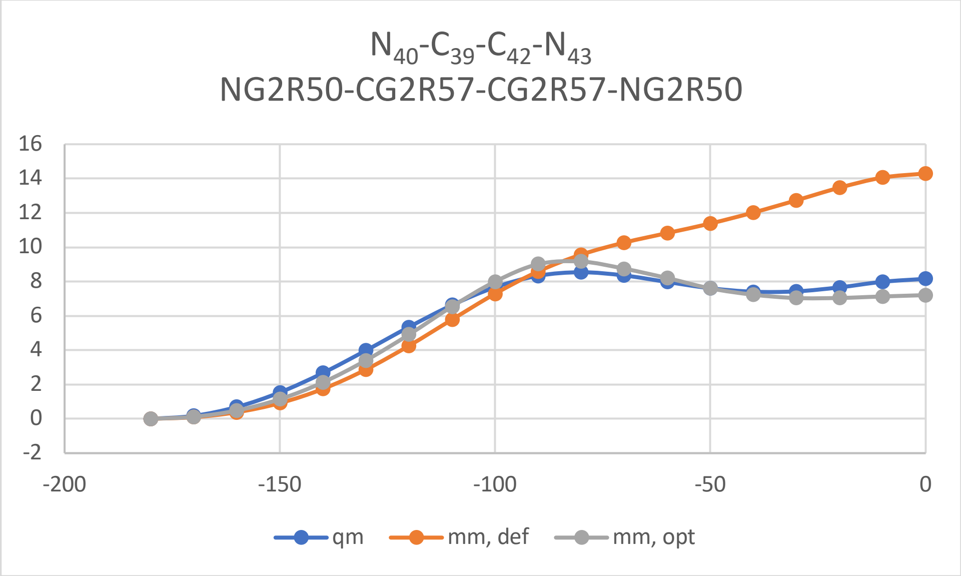

Figure S8. Potential-energy curve for the $N_{40}C_{39}C_{42}N_{43}$ angle between -200° and 0°, calculated with MP2/6-31G(d) (blue) and with default CGengFF (orange) or with our optimized forcefield (gray). The Y axis shows the energy (in kcal/mol) relative to the lowest-energy conformer.

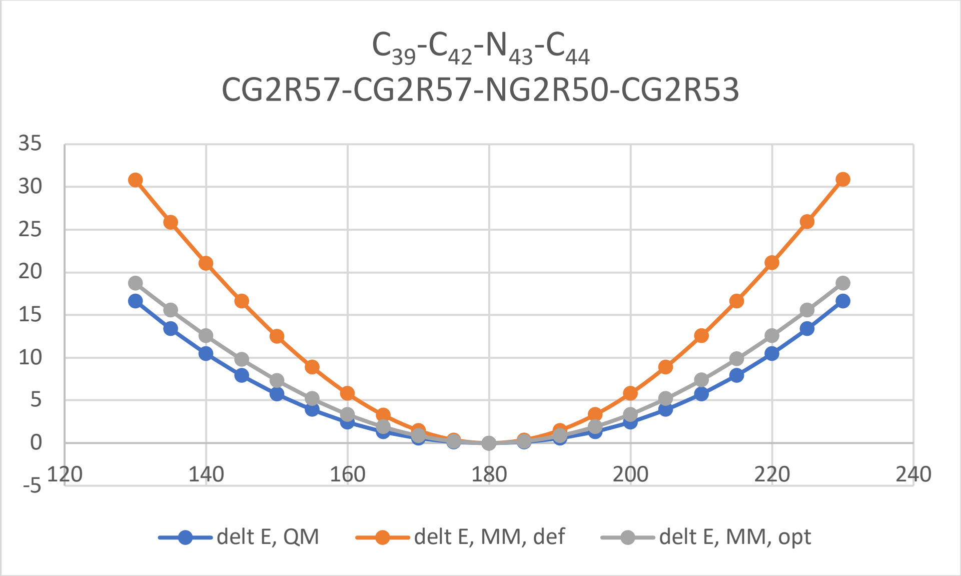

Figure S9. Potential-energy curve for the $C_{39}C_{42}N_{43}C_{44}$ angle between 120° and 240°, calculated with MP2/6-31G(d) (blue) and with default CGengFF (orange) or with our optimized forcefield (gray). The Y axis shows the energy (in kcal/mol) relative to the lowest-energy conformer.

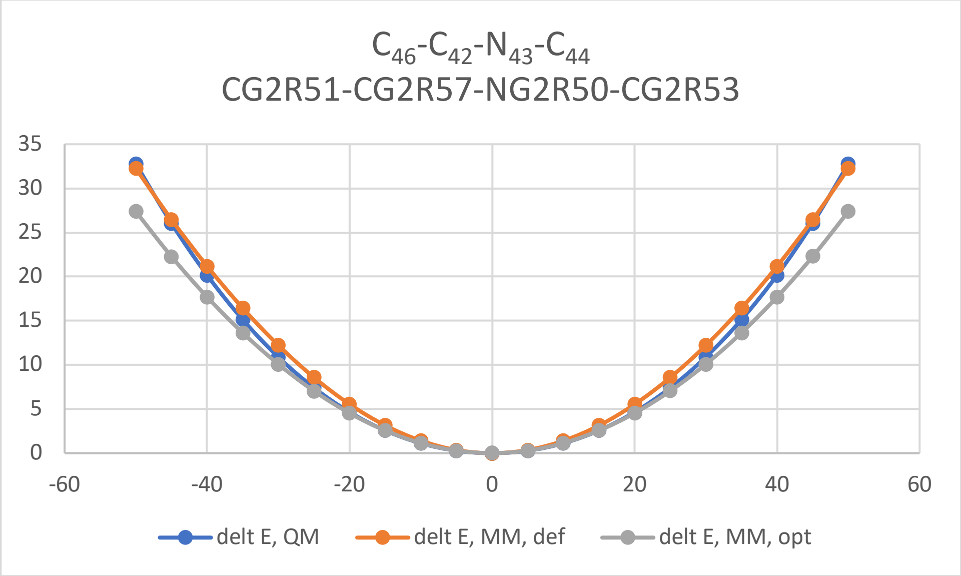

Figure S10. Potential-energy curve for the $C_{46}C_{42}N_{43}C_{44}$ angle between -60° and 60°, calculated with MP2/6-31G(d) (blue) and with default CGengFF (orange) or with our optimized forcefield (gray). The Y axis shows the energy (in kcal/mol) relative to the lowest-energy conformer.

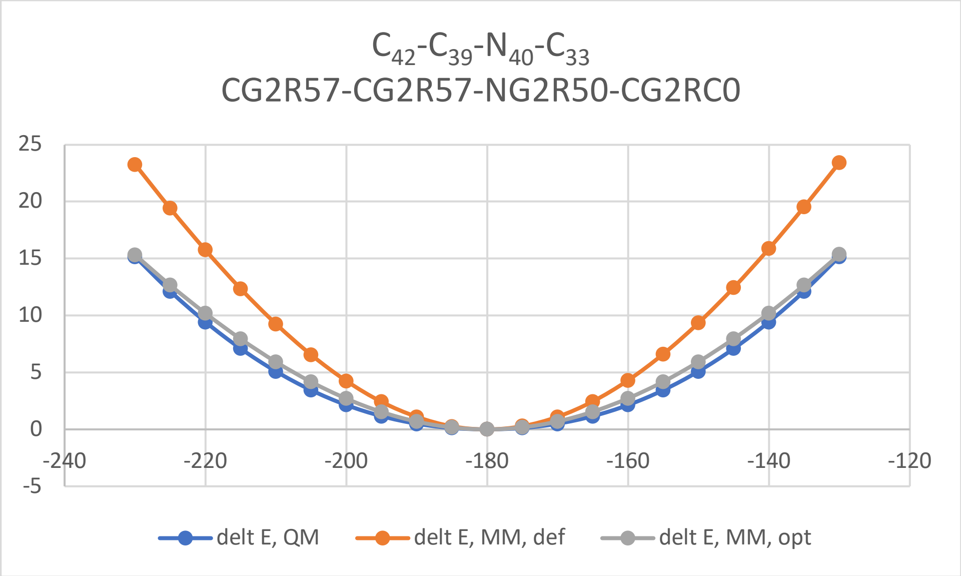

Figure S11. Potential-energy curve for the $C_{42}C_{39}N_{40}C_{33}$ angle between -240° and -120°, calculated with MP2/6-31G(d) (blue) and with default CGengFF (orange) or with our optimized forcefield (gray). The Y axis shows the energy (in kcal/mol) relative to the lowest-energy conformer.

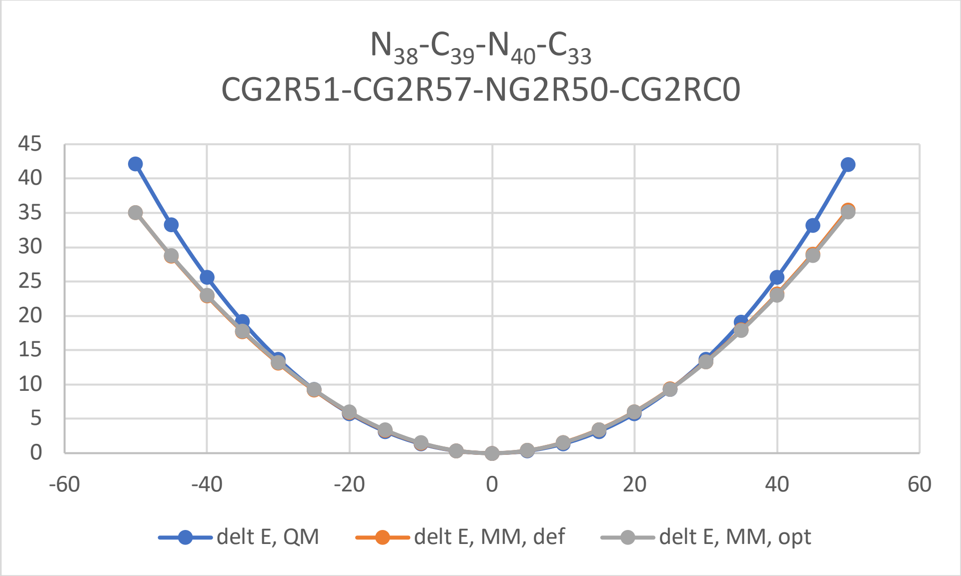

Figure S12. Potential-energy curve for the $N_{38}C_{39}N_{40}C_{33}$ angle between -60° and 60°, calculated with MP2/6-31G(d) (blue) and with default CGengFF (orange) or with our optimized forcefield (gray). The Y axis shows the energy (in kcal/mol) relative to the lowest-energy conformer.

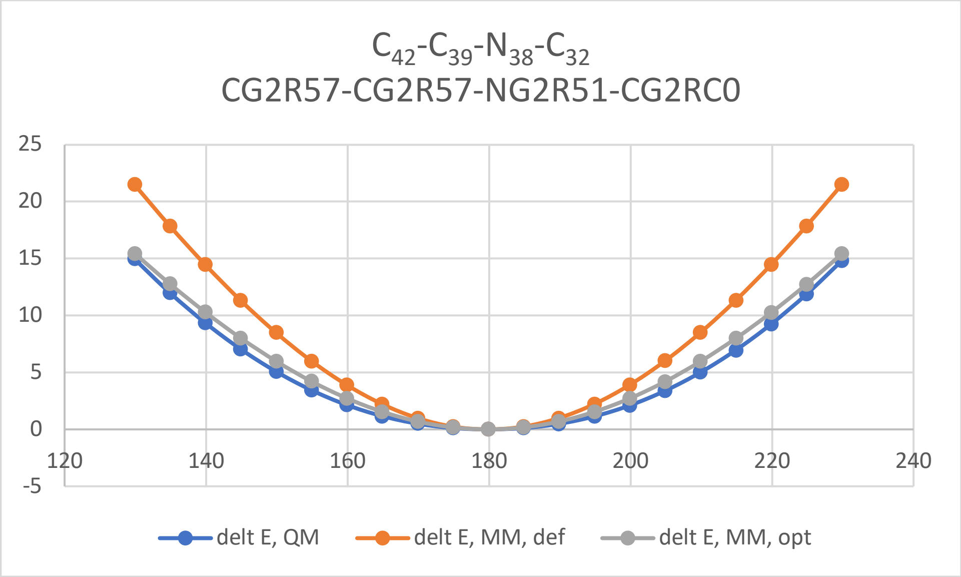

Figure S13. Potential-energy curve for the $C_{42}C_{39}N_{38}C_{32}$ angle between 120° and 240°, calculated with MP2/6-31G(d) (blue) and with default CGengFF (orange) or with our optimized forcefield (gray). The Y axis shows the energy (in kcal/mol) relative to the lowest-energy conformer.

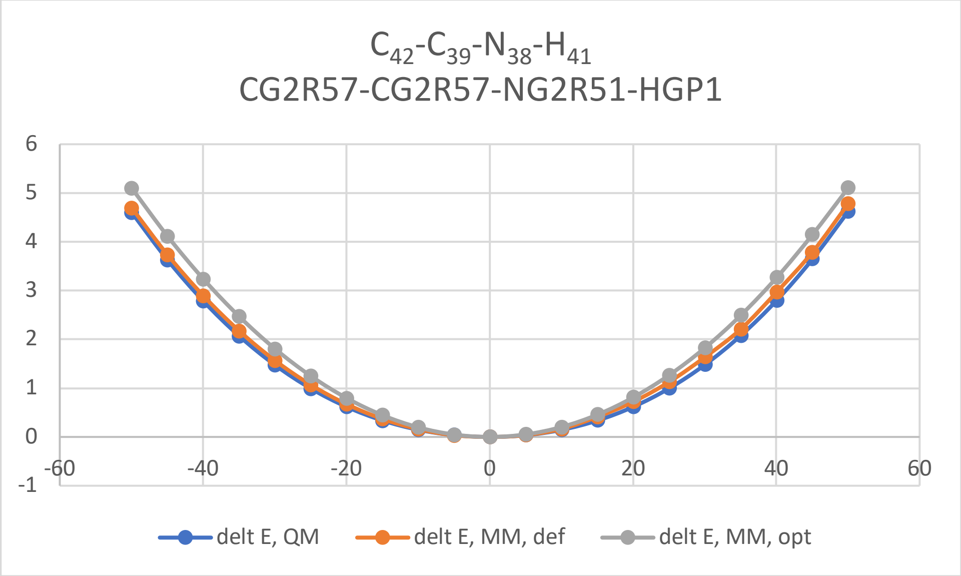

Figure S14. Potential-energy curve for the $C_{42}C_{39}N_{38}H_{41}$ angle between -60° and 60°, calculated with MP2/6-31G(d) (blue) and with default CGengFF (orange) or with our optimized forcefield (gray). The Y axis shows the energy (in kcal/mol) relative to the lowest-energy conformer.

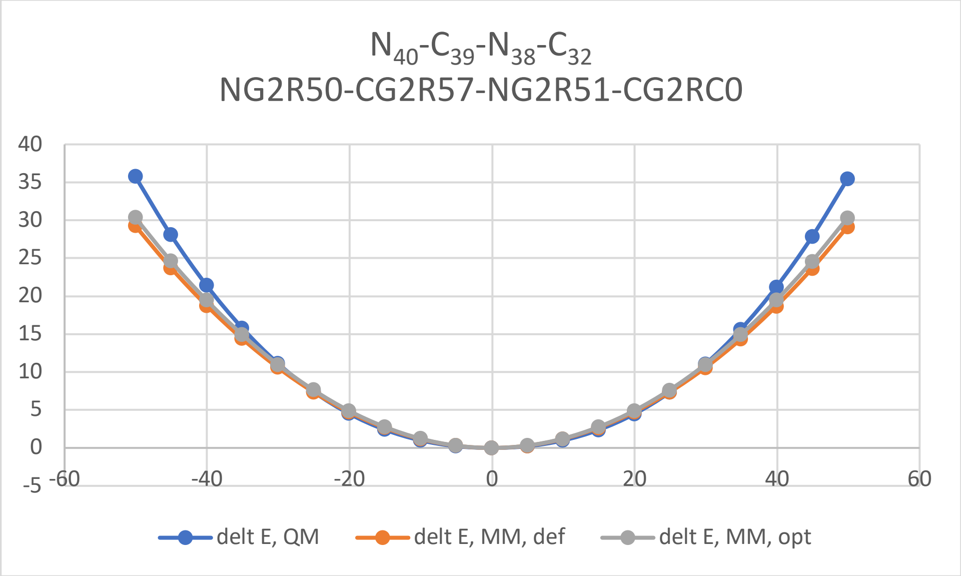

Figure S15. Potential-energy curve for the $N_{40}C_{39}N_{38}C_{32}$ angle between -60° and 60°, calculated with MP2/6-31G(d) (blue) and with default CGengFF (orange) or with our optimized forcefield (gray). The Y axis shows the energy (in kcal/mol) relative to the lowest-energy conformer.

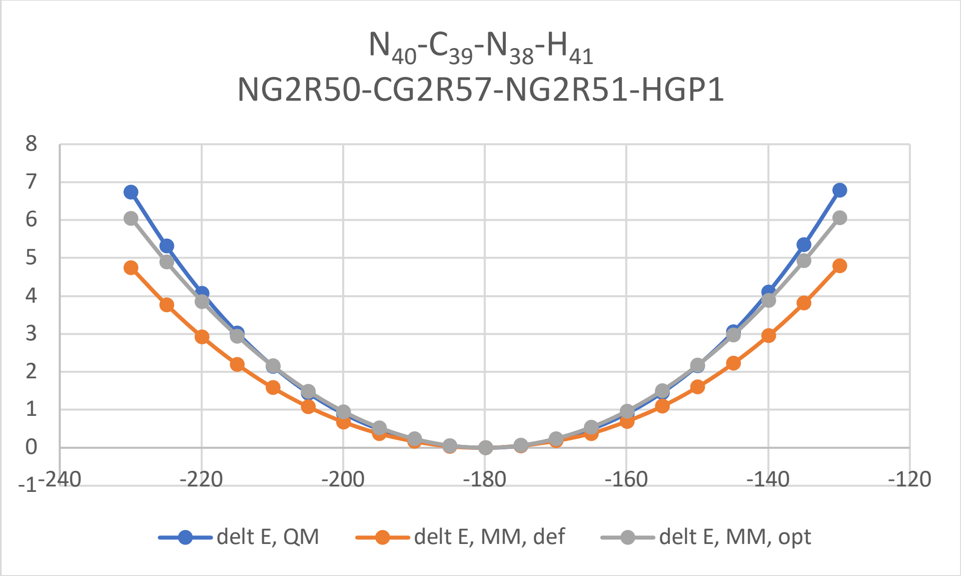

Figure S16. Potential-energy curve for the $N_{40}C_{39}N_{38}H_{41}$ angle between -240° and -120°, calculated with MP2/6-31G(d) (blue) and with default CGengFF (orange) or with our optimized forcefield (gray). The Y axis shows the energy (in kcal/mol) relative to the lowest-energy conformer.

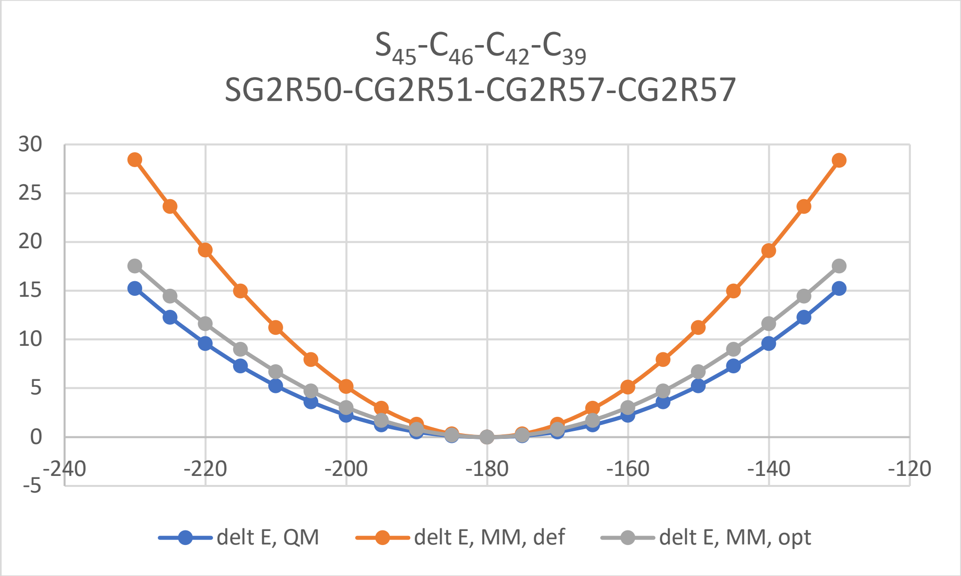

Figure S17. Potential-energy curve for the $S_{45}C_{46}C_{42}C_{39}$ angle between -240° and -120°, calculated with MP2/6-31G(d) (blue) and with default CGengFF (orange) or with our optimized forcefield (gray) by scanning. The Y axis shows the energy (in kcal/mol) relative to the lowest-energy conformer.

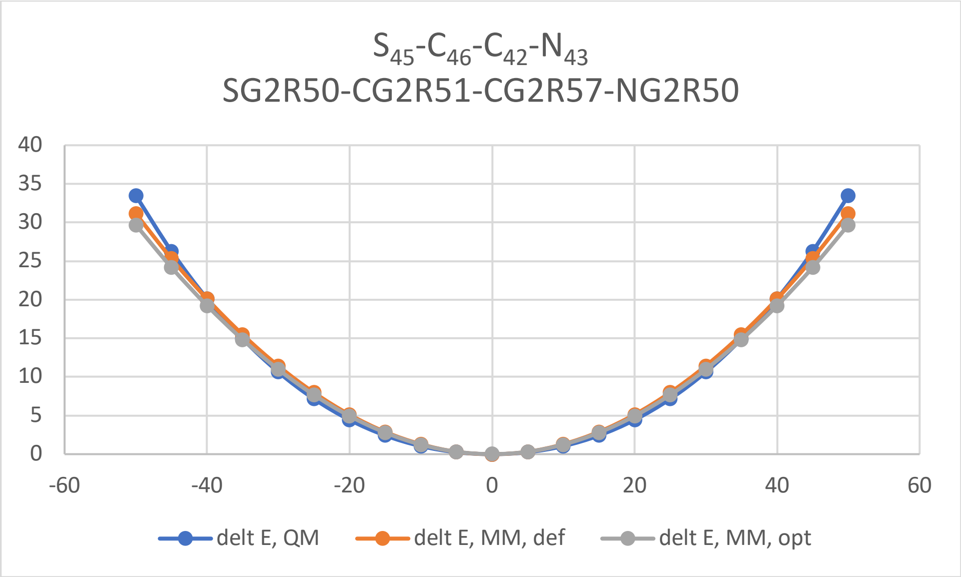

Figure S18. Potential-energy curves for the $S_{45}C_{46}C_{42}N_{43}$ angle between -60° and 60°, calculated with MP2/6-31G(d) (blue) and with default CGengFF (orange) or with our optimized forcefield (gray). The Y axis shows the energy (in kcal/mol) relative to the lowest-energy conformer.

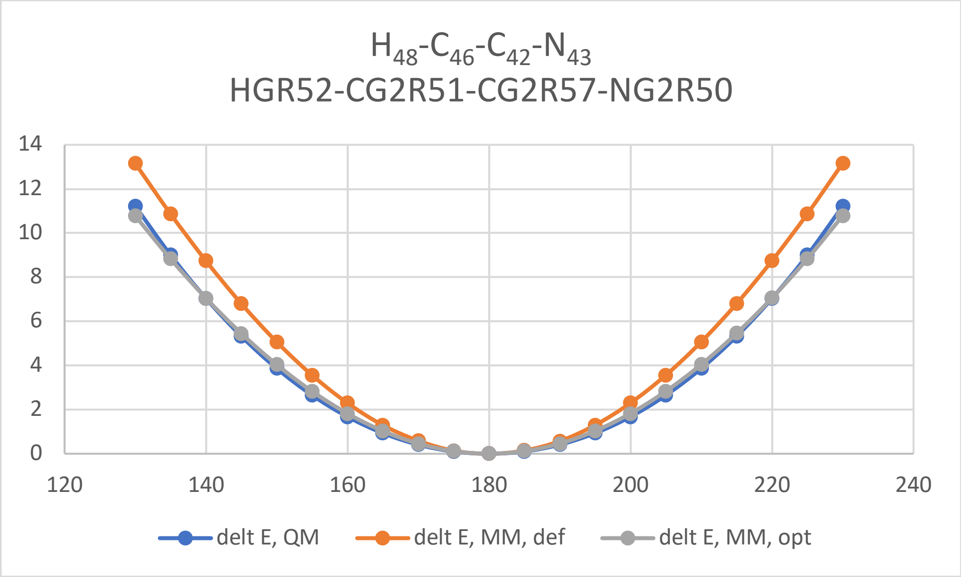

Figure S19. Potential-energy curve for the $H_{48}C_{46}C_{42}N_{43}$ angle between 120° and 240°, calculated with MP2/6-31G(d) (blue) and with default CGengFF (orange) or with our optimized forcefield (gray). The Y axis shows the energy (in kcal/mol) relative to the lowest-energy conformer.

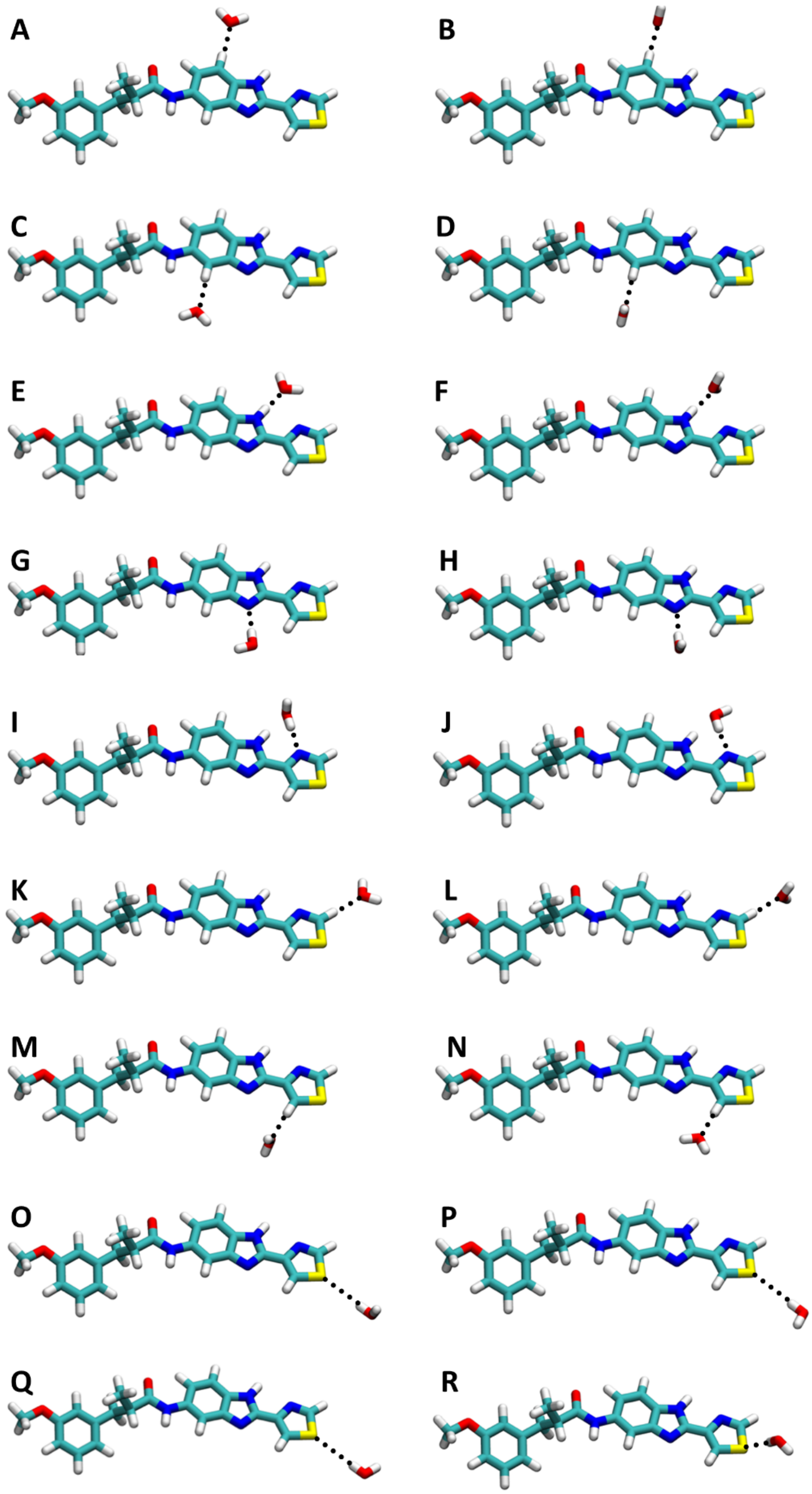

Figure S20. Ab-initio optimized geometries of RY785-water complexes used in the calibration of our molecular-mechanics forcefield for RY785.

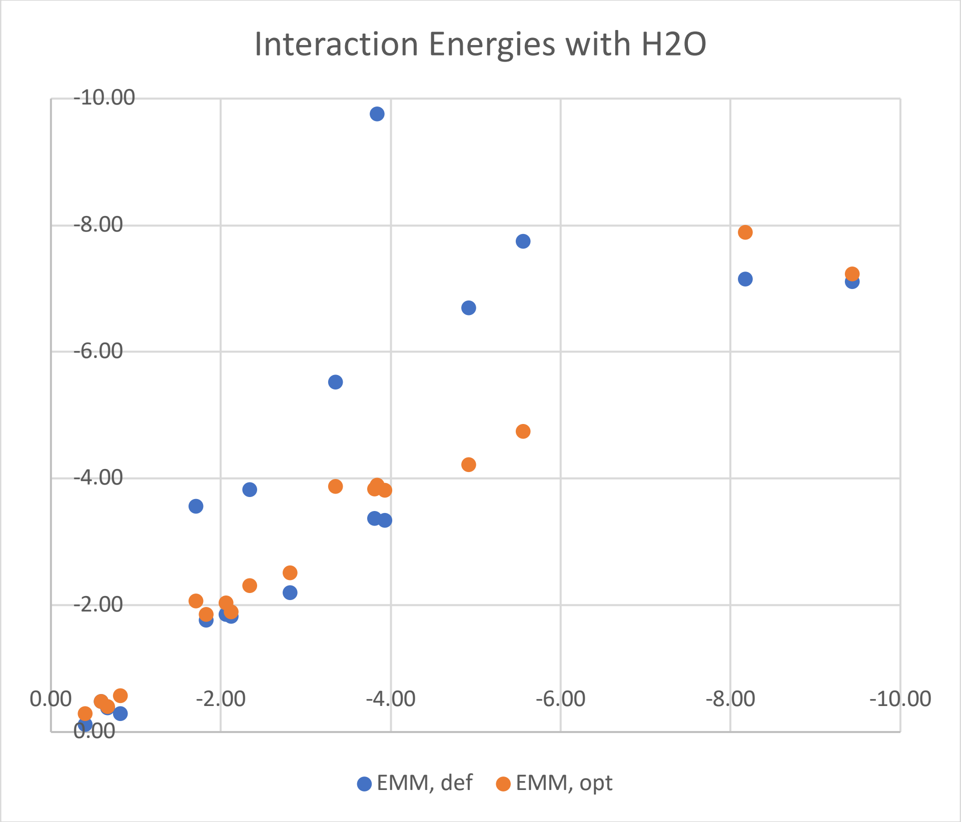

Figure S21. Interaction energies (in kcal/mol) for the RY785-water complexes shown in Fig. S20. QM data (Y-axis) are correlated with MM values calculated with default CGenFF (blue) and with our optimized forcefield (orange).

Table S1. Interaction distances (in Å) and Interaction energies (in kcal/mol) for the RY785-water complexes shown in Fig. S20. QM data are compared with MM values calculated with default CGenFF and with our optimized forcefield.

Complex	E_{QM}	r_{QM}	$E_{MM, def}$	$r_{MM, def}$	$E_{MM, opt}$	$r_{MM, opt}$
**A**	-1.83	2.58	-1.76	2.60	-1.86	2.59
**B**	-2.12	2.51	-1.82	2.60	-1.90	2.60
**C**	-1.71	2.61	-3.56	2.53	-2.07	2.64
**D**	-2.34	2.47	-3.83	2.51	-2.32	2.62
**E**	-4.92	1.99	-6.70	1.83	-4.22	1.92
**F**	-3.35	2.14	-5.53	1.87	-3.88	1.94
**G**	-8.17	2.06	-7.15	1.88	-7.89	1.87
**H**	-9.44	2.04	-7.11	1.88	-7.24	1.88
**I**	-5.56	2.09	-7.75	1.85	-3.90	1.97
**J**	-3.84	2.07	-9.76	1.83	-4.75	1.95
**K**	-3.81	2.32	-3.38	2.23	-3.84	2.22
**L**	-3.93	2.30	-3.34	2.24	-3.82	2.22
**M**	-2.06	2.31	-1.86	2.26	-2.04	2.25
**N**	-2.81	2.22	-2.20	2.22	-2.51	2.21
**O**	-0.67	2.86	-0.38	2.60	-0.40	2.62
**P**	-0.59	2.92	-0.49	2.59	-0.48	2.60
**Q**	-0.40	2.94	-0.12	2.66	-0.29	2.67
**R**	-0.82	2.80	-0.29	2.58	-0.37	2.57
